# Adolescent health in the Eastern Mediterranean Region: findings from the global burden of disease 2015 study

**DOI:** 10.1007/s00038-017-1003-4

**Published:** 2017-08-03

**Authors:** GBD 2015 Eastern Mediterranean Region Adolescent Health Collaborators, Peter Azzopardi, Karly Cini, Elissa Kennedy, Susan Sawyer, Charbel El Bcheraoui, Raghid Charara, Ibrahim Khalil, Maziar Moradi-Lakeh, Michael Collison, Rima A. Afifi, Jamela Al-raiby, Kristopher J. Krohn, Farah Daoud, Adrienne Chew, Ashkan Afshin, Kyle J. Foreman, Nicholas J. Kassebaum, Michael Kutz, Hmwe H. Kyu, Patrick Liu, Helen E. Olsen, Alison Smith, Jeffrey D. Stanaway, Haidong Wang, Johan Ärnlöv, Aliasghar Ahmad Kiadaliri, Khurshid Alam, Deena Alasfoor, Raghib Ali, Reza Alizadeh-Navaei, Rajaa Al-Raddadi, Khalid A. Altirkawi, Nelson Alvis-Guzman, Nahla Anber, Carl Abelardo T. Antonio, Palwasha Anwari, Al Artaman, Hamid Asayesh, Suzanne L. Barker-Collo, Neeraj Bedi, Ettore Beghi, Derrick A. Bennett, Isabela M. Bensenor, Zulfiqar A. Bhutta, Zahid A. Butt, Carlos A. Castañeda-Orjuela, Ferrán Catalá-López, Fiona J. Charlson, Hadi Danawi, Diego De Leo, Louisa Degenhardt, Donna Denno, Kebede Deribe, Don C. Des Jarlais, Subhojit Dey, Samath D. Dharmaratne, Shirin Djalalinia, Holly E. Erskine, Seyed-Mohammad Fereshtehnejad, Alize J. Ferrari, Florian Fischer, Tsegaye Tewelde Gebrehiwot, Johanna M. Geleijnse, Philimon N. Gona, Harish Chander Gugnani, Rajeev Gupta, Randah Ribhi Hamadeh, Samer Hamidi, Josep Maria Haro, Roderick J. Hay, Stephen J. C. Hearps, Delia Hendrie, Peter J. Hotez, Guoqing Hu, Jost B. Jonas, André Karch, Seyed M. Karimi, Amir Kasaeian, Seifu Kebede, Andre Pascal Kengne, Ejaz Ahmad Khan, Ardeshir Khosravi, Jagdish Khubchandani, Yoshihiro Kokubo, Jacek A. Kopec, Soewarta Kosen, Heidi J. Larson, Anders Larsson, Janet L. Leasher, Janni Leung, Yongmei Li, Paulo A. Lotufo, Raimundas Lunevicius, Hassan Magdy Abd El Razek, Reza Majdzadeh, Azeem Majeed, Peter Memiah, Ziad A. Memish, Walter Mendoza, Francis Apolinary Mhimbira, Ted R. Miller, Philip B. Mitchell, Lorenzo Monasta, Carla Makhlouf Obermeyer, In-Hwan Oh, Bolajoko Olubukunola Olusanya, Alberto Ortiz, Eun-Kee Park, Matti Parry, David M. Pereira, Michael Robert Phillips, Farshad Pourmalek, Mostafa Qorbani, Amir Radfar, Anwar Rafay, Vafa Rahimi-Movaghar, Rajesh Kumar Rai, Saleem M. Rana, David Laith Rawaf, Salman Rawaf, Nicola Reavley, Andre M. N. Renzaho, Satar Rezaei, Mohammad Sadegh Rezai, Diego Rios-Zertuche, Gholamreza Roshandel, Dietrich Rothenbacher, Mahdi Safdarian, Sare Safi, Saeid Safiri, Mohammad Ali Sahraian, Payman Salamati, Abdallah M. Samy, Juan Ramon Sanabria, Damian Santomauro, Benn Sartorius, David C. Schwebel, Soraya Seedat, Sadaf G. Sepanlou, Tesfaye Setegn, Amira Shaheen, Masood Ali Shaikh, Rahman Shiri, Inga Dora Sigfusdottir, Jasvinder A. Singh, Badr H. A. Sobaih, Chandrashekhar T. Sreeramareddy, Rizwan Suliankatchi Abdulkader, Arash Tehrani-Banihashemi, Mohamad-Hani Temsah, Abdullah Sulieman Terkawi, Alan J. Thomson, Marcello Tonelli, Roman Topor-Madry, Bach Xuan Tran, Kingsley Nnanna Ukwaja, Olalekan A. Uthman, Tommi Vasankari, Narayanaswamy Venketasubramanian, Vasiliy Victorovich Vlassov, Stein Emil Vollset, Elisabete Weiderpass, Robert G. Weintraub, Andrea Werdecker, Harvey A. Whiteford, Yuichiro Yano, Mehdi Yaseri, Naohiro Yonemoto, Mustafa Z. Younis, Chuanhua Yu, Aisha O. Jumaan, Theo Vos, Simon I. I. Hay, Mohsen Naghavi, George C. Patton, Christopher J. L. Murray, Ali H. Mokdad

**Affiliations:** Institute for Health Metrics and Evaluation, University of Washington, Seattle, WA USA

**Keywords:** Adolescent health, Burden of disease, Eastern Mediterranean Region

## Abstract

**Objectives:**

The 22 countries of the East Mediterranean Region (EMR) have large populations of adolescents aged 10–24 years. These adolescents are central to assuring the health, development, and peace of this region. We described their health needs.

**Methods:**

Using data from the Global Burden of Disease Study 2015 (GBD 2015), we report the leading causes of mortality and morbidity for adolescents in the EMR from 1990 to 2015. We also report the prevalence of key health risk behaviors and determinants.

**Results:**

Communicable diseases and the health consequences of natural disasters reduced substantially between 1990 and 2015. However, these gains have largely been offset by the health impacts of war and the emergence of non-communicable diseases (including mental health disorders), unintentional injury, and self-harm. Tobacco smoking and high body mass were common health risks amongst adolescents. Additionally, many EMR countries had high rates of adolescent pregnancy and unmet need for contraception.

**Conclusions:**

Even with the return of peace and security, adolescents will have a persisting poor health profile that will pose a barrier to socioeconomic growth and development of the EMR.

**Electronic supplementary material:**

The online version of this article (doi:10.1007/s00038-017-1003-4) contains supplementary material, which is available to authorized users.

## Introduction

The World Health Organization’s Eastern Mediterranean Region (EMR) is an administrative region of 22 countries (Table 1) that while rich in natural resources, has marked country-level variation in socioeconomic wealth (ranging from $US 549.3 per capita in Somalia to $US 73,653.4 per capita in Qatar), health system capacities and health coverage (Blair et al. 2014; Mandil et al. 2013; WHO 2017b). Many countries in the EMR have recently experienced social and political instabilities, civil unrest, war, and mass displacement of people (Mokdad et al. 2016a). As a result, health in many EMR countries has failed to improve in recent years (Mokdad et al. 2016a, 2014). As other papers in this series highlight, there is now an increasing burden of many preventable health problems including HIV, mental health disorders, and intentional injury (GBD 2015 Eastern Mediterranean Region HIV/AIDS Collaborators and Mokdad 2017; GBD 2015 Eastern Mediterranean Region Intentional Injuries Collaborators and Mokdad 2017; GBD 2015 Eastern Mediterranean Region Mental Health Collaborators and Mokdad 2017). There is a risk that without urgent action, the health status of this region will only deteriorate further, with both regional and global consequences for health, social stability, and economic development.

**Table 1:** Eastern Mediterranean region: Countries, adolescent population and socioeconomic development, 1990–2015 (World Bank, Global Burden of Disease Study 2015, Eastern Mediterranean Countries, 1990–2015)

Country	Proportion of country population aged 10–24 year in % (*n*, number of adolescents in each country)	Proportion of the EMR adolescent population (%)	GDP per capita ($US)	Socio-demographic index (SDI)		
				1990	2015	SDI level
Afghanistan	34.8% (11,356,556)	6.2	594.3	0.1440	0.2888	Low
Bahrain	21.7% (296,971)	0.2	22,600.2	0.5969	0.7764	High-middle
Djibouti	30.5% (271,064)	0.1	1945.1	0.3228	0.4615	Low-middle
Egypt	26.9% (24,492,800)	13.3	3614.7	0.4409	0.6191	Middle
Iran	22.8% (17,992,150)	9.8	–	0.4600	0.7154	High-middle
Iraq	31.2% (11,348,292)	6.2	4943.8	0.3997	0.5756	Middle
Jordan	29.9% (2,263,213)	1.2	4940.0	0.4967	0.6949	High-middle
Kuwait	19.1% (745,077)	0.4	29,300.6	0.6911	0.8624	High
Lebanon	28.5% (1,643,663)	0.9	8047.6	0.5698	0.7547	High-middle
Libya	25.0% (1,574,514)	0.9	–	0.4747	0.6430	Middle
Morocco	26.0% (8,932,361)	4.8	2878.2	0.3347	0.4959	Low-middle
Oman	21.4% (960,174)	0.5	15,550.7	0.4089	0.7301	High-middle
Pakistan	30.2% (57,088,761)	31	1434.7	0.2786	0.4676	Low-middle
Palestine	33.6% (1,569,806)	0.9	–	0.4229	0.5670	Middle
Qatar	19.3% (429,261)	0.2	73,653.4	0.6162	0.8045	High-middle
Saudi Arabia	24.4% (7,683,094)	4.2	20,481.7	0.5245	0.7593	High-middle
Somalia	32.7% (3,545,571)	1.9	549.3	0.1158	0.1506	Low
Sudan	32.1% (12,950,382)	7.0	2414.7	0.2667	0.4282	Low-middle
Syria	32.4% (6,032,616)	3.3	–	0.3881	0.5790	Middle
Tunisia	22.6% (2,546,994)	1.4	3872.5	0.4503	0.6515	Middle
United Arab Emirates	16.6% (1,516,072)	0.8	40,438.8	0.6324	0.8747	High
Yemen	34.2% (9,191,689)	5.0	1406.3	0.1329	0.4080	Low-middle
Total	28.4% (184,431,081)	100	–	–	–	–

This table details the 22 countries in the East Mediterranean region. It provides the population of adolescents and contribution of each country to the total adolescent population in the Eastern Mediterranean Region. It also provides the overall country-level GDP in 2015 and SDI in 1990 and 2015. Dashes indicate data are unavailable

Adolescence is increasingly understood as a key developmental stage for assuring health across the course of one’s life, and as such, provides significant opportunities to improve population health in the EMR (Patton et al. 2016; The World Bank 2006). Firstly, adolescents represent more than a quarter of the population in the EMR, and their health needs are likely to be distinct from children and adults. Conflict and civil unrest (which have been a feature of many countries in the EMR) have a large impact on the health of young people, both acutely (through high rates of mortality and morbidity due to violence) but also in the longer term (including mental health disorder and poor sexual and reproductive health) (Viner et al. 2012). Secondly, many health risks typically emerge during adolescence including those for non-communicable diseases (NCDs) such as substance use, overweight, and physical inactivity. Given that NCDs are now the leading cause of poor health in the EMR, there is a potential to intervene before harms arise (Mokdad et al. 2016b). Thirdly, adolescents are critical to driving socioeconomic development (The World Bank 2006). Poor physical health and mental health are barriers to participation in education and employment, as are policies and systems that do not enable equitable access. Finally, in their role as current and future parents, the health of adolescents has significant implications for the next generation (Patton et al. 2016).

To date, the health problems and health risks of adolescents in the EMR have been inadequately described (Alaovie et al. 2017). This is a significant barrier to developing comprehensive policies that address adolescent health and to measuring the impact of any investments made. This paper aims to report the health profile for adolescents living in the EMR.

## Methods

We framed our study around the conceptual framework defined by the Lancet Commission of Adolescent Health and Wellbeing (hereafter referred to as the Commission) (Patton et al. 2016). Health needs included: health outcomes (mortality, non-fatal diseases, and injuries); health risks (behaviors and states that carry risk for poor health in and beyond adolescence); and determinants of health (such as education and employment). Adolescence was defined as 10–24 years, as these years encompass the important biological, neurocognitive, and social role transitions that typically define adolescence (Mokdad et al. 2016b; Patton et al. 2016; Sawyer et al. 2012). Where possible, we report age-disaggregated data for young adolescents (10–14 years), older adolescents (15–19 years), and young adults (20–24 years) (Patton et al. 2016).

Data are drawn from the Global Burden of Disease Study 2015 (GBD 2015) as this provides a complete set of comparable health estimates for 195 countries, including all those in the EMR. Methods are described in detail elsewhere (GBD DALYs Hale Collaborators 2016; GBD Disease Injury Incidence and Prevalence Collaborators 2016; GBD Mortality and Causes of Death Collaborators 2016; GBD SDG Collaborators 2016), but briefly, GBD 2015 includes a comprehensive and systematic analysis of 249 causes of death, 310 causes of disease and injury, and 79 behavioral and environmental health risks. GBD 2015 has four levels of causes that are mutually exclusive. Level one has three causes: type I conditions (communicable, maternal, neonatal, and nutritional disorders); non-communicable diseases; and injuries. Level two has 21 causes, while levels three and four consist of all disaggregated causes. For this analysis we report causes at level four. GBD is based on the best available primary data and employs a series of disease models to harmonize health estimates and fill data gaps. Each step of the estimation process of GBD 2015 has been documented, as well as data sources, in accordance with Guidelines for Accurate and Transparent Health Estimates Reporting (GATHER). For this analysis, we accessed data in 5-year age bands, for males and females, from 1990 to 2015 in 5-year time slices.

Mortality is reported as all-cause and cause-specific rates per 100,000 (GBD Mortality and Causes of Death Collaborators 2016). Non-fatal diseases and injuries are reported as years lived with disability (YLDs), a metric which incorporates prevalence of disease, duration, and its severity (using disease weights) (GBD Disease Injury Incidence and Prevalence Collaborators 2016). As a summary measure of population health, we also report disability-adjusted life-years (DALYs), the sum of years of healthy life lost due to premature mortality (YLLs), and years of life lived with disability (YLDs) (GBD DALYs Hale Collaborators 2016). For these estimates, we report 95% uncertainty estimates, which are distinct from confidence intervals in that they represent uncertainty derived from sampling, model estimation, and model specification (GBD DALYs Hale Collaborators 2016).

In addition to region-level estimates, we also report country-specific DALY estimates and the prevalence of three key health risks and four determinants (aligned with the conceptual framework from the Commission and data availability) to help prioritize country-specific actions (Patton et al. 2016). Data for health risks were sourced from GBD 2015. Tobacco smoking was defined as current daily smoked tobacco use (GBD Tobacco Collaborators 2017). Overweight was defined using the International Obesity Task Force age and gender specific cut-offs, equivalent to BMI ≥25 kg/m^2^ at age 18 (Cole and Lobstein 2012). This definition includes those who are obese. Binge drinking was defined as having consumed 60 grams of alcohol on a single occasion for males and 48 grams of alcohol on a single occasion for females in the last 12 months. With respect to determinants, adolescent fertility rate (live births per 1000 15- to 19-year-old females) and mean years of educational attainment for 15- to 24-year olds were sourced from GBD 2015 (GBD SDG Collaborators 2016). Unmet need for contraception (15- to 24-year-old females currently married or in union and not wanting to become pregnant within the next two years, who report not using any method of contraception) was sourced from a review DHS and MICS surveys available in the EMR (data were collected from 2009 to 2014) (Patton et al. 2016). Youth unemployment data, defined as the percentage of 15- to 24-year olds without work but available for and seeking employment, were obtained from the International Labor Organization modeled estimates for 2013 (Patton et al. 2016).

We reported observed estimates for the region. We additionally report *expected* DALYs for each country based on the level of socioeconomic development. Expected DALYs were estimated using the Socio-demographic Index (SDI) which is based on income per capita, average educational attainment for ages 15 or older, and the total fertility rate (GBD SDG Collaborators 2016). SDI is reported as a continuous variable from 0 (lowest) to 1 (highest), and as quintiles, as shown in Table 1. GBD 2015 has estimated the relationship between SDI and each cause of DALYs using spline regressions, with these regressions then used to estimate expected DALYs at each level of SDI (GBD DALYs Hale Collaborators 2016).

## Results

### Mortality

All-cause mortality rates for adolescents in the EMR ranged from 63.3 per 100,000 for females aged 10–14 years to 253.2 per 100,000 for males aged 20–24 years in 2015 (e-Figure 1, panel A). Males had a higher overall mortality rate than females. The risk of mortality for males aged 15–24 years had increased over recent years compared to what was otherwise an overall trend of reduction in mortality. Table 2 details the leading causes of mortality by sex. The most striking transition in mortality cause over time was the reduction in deaths due to natural disaster and communicable diseases, and the emergence of mortality due to injuries (especially in the context of war) and NCDs. In 1990 and 2005 natural disaster ranked as a leading cause of mortality for adolescents, whereas in 2015 it was no longer a leading cause of death for adolescents. In 2015, war and legal interventions (law enforcement) was the leading cause of death for adolescents of both sexes, representing 27.7% (14.2–38.4) of deaths amongst male 20- to 24-year olds and 7.2% (3.1–10.9) amongst female 20- to 24-year olds. For males, injuries (unintentional injuries, self-harm and violence) were the predominant causes of mortality across adolescence, and communicable diseases an important cause for 10- to 14-year olds. The leading cause of mortality for females included injuries; however, communicable and maternal conditions were also leading causes, with NCDs emerging as an important cause amongst older female adolescents.

**Table 2:** Leading contributors to poor health (mortality) for adolescents in the Eastern Mediterranean Region, in 1990, 2005, 2015

	Top ten causes of mortality in females and males						
	Females						
	1990			2005		2015	
	Rank	Cause	Proportion	Cause	Proportion	Cause	Proportion
10–14 years	1	***Natural disaster***	***8.0% (3.2–12.6)***	***Natural disaster***	***12.8% (7.4–18)***	***War and legal intervention***	***9.5% (4.2–14.2)***
	2	*Diarrheal diseases*	*5.8% (4.1–7.8)*	*Typhoid fever*	*5.3% (2.9–8.9)*	*Lower respiratory infect.*	*5.6% (3.6–7.3)*
	3	*Lower respiratory infect.*	*5.7% (3.5–7.4)*	*Lower respiratory infect.*	*4.7% (3.2–6.1)*	*Typhoid fever*	*5.2% (2.9–8.7)*
	4	*Measles*	*5.3% (1.5–12.7)*	*Diarrheal diseases*	*4.6% (3.2–6.7)*	**Congenital heart**	**4.8% (3.3–6.5)**
	5	*Tuberculosis*	*4.5% (2.2–67)*	*Tuberculosis*	*4.4% (2.5–6.4)*	***Drowning***	***4.3% (2.7–5.9)***
	6	***Drowning***	***4.3% (2.5–6.4)***	***Drowning***	***4.1% (2.6–5.8)***	*Tuberculosis*	*4.0% (2.3–6)*
	7	*Typhoid fever*	*4.3% (2 3–7.5)*	**Congenital heart**	**3.9% (2.6–5.8)**	*Diarrheal diseases*	*3.9% (2.6–5.9)*
	8	***Motor vehicle road inj.***	***3.2% (2.4–4.2)***	***Motor vehicle road inj.***	3.5% (2.8–4.2)	***Motor vehicle road inj.***	***3.6% (2.9–4.5)***
	9	**Hemorrhagic stroke**	**3.1% (2.5–3.9)**	*Malaria*	*2.8% (0.9–7.2)*	***Other unintentional***	***2.7% (1.7–4.1)***
	10	**Congenital heart**	**3.1% (2–5.2)**	*Measles*	*2.4% (0.6–6.7)*	*Malaria*	*2.6% (0.7–7.6)*
15–19 years	1	***Natural disaster***	***5.8% (2.2–9.3)***	***Natural disaster***	***8.1% (4.4–11.5***	***War and legal intervention***	***10.1% (4.4–15.5)***
	2	*Tuberculosis*	*5.8% (3.3–8.7)*	*Maternal hemorrhage*	*5.4% (3.7–7.2)*	*Tuberculosis*	*4.7% (2.8–6.7)*
	3	*Maternal hemorrhage*	*5.5% (3.8–7.7)*	*Tuberculosis*	*5.2% (3.2–7.2)*	*Maternal hemorrhage*	*3.8% (2.3–5.6)*
	4	*Diarrheal diseases*	*4.2% (3–5.7)*	*Maternal hypertension*	*3.6% (2.4–5)*	***Motor vehicle road inj.***	***3.5% (2.8–4.4)***
	5	*Maternal hypertension*	*3.8% (2.4–5.7)*	***Motor vehicle road inj.***	***3.6% (2.9–4.4)***	*Maternal hypertension*	*3.4% (2–5.3)*
	6	***Drowning***	***3.4% (2–5.2)***	*Diarrheal diseases*	*3.5% (2.4–4.9)*	***Drowning***	***3.4% (2–4.8)***
	7	**Ischemic heart disease**	**3.0% (2.5–3.6)**	***Drowning***	***3.4% (2–4.7)***	*Malaria*	*3.3% (1.5–6.3)*
	8	***Motor vehicle road inj.***	***2.9% (2.3–3.7)***	***Self-harm***	***3.4% (2.5–6.3)***	***Self-harm***	***2.9% (2.1–6.3)***
	9	***Self-harm***	***2.9% (1.9–5.3)***	**Ischemic heart disease**	**2.9% (2.4–3.5)**	*Diarrheal diseases*	*2.9% (1.9–4.2)*
	10	**Hemorrhagic stroke**	**2.8% (2.3–3.4)**	*Malaria*	*2.9% (1.5–5.1)*	**Ischemic heart disease**	**2.8% (2.3–3.5)**
20–24 years	1	*Maternal hemorrhage*	*8.2% (5.8–11)*	*Maternal hemorrhage*	*7.7% (5.7–10.2)*	***War and legal intervention***	***7.2% (3.1–10.9)***
	2	*Tuberculosis*	*7.1% (4.4–10.7)*	*Tuberculosis*	*6.2% (4–8.5)*	*Tuberculosis*	*5.9% (3.7–8.1)*
	3	*Maternal hypertension*	*5.6% (3.7––8.2)*	***Natural disaster***	***5.9% (3.2–8.5)***	*Maternal hemorrhage*	*5.6% (3.7–8.1)*
	4	***Natural disaster***	***4.1% (1.5–6.6)***	*Maternal hypertension*	**5.4% (3.8–7.4)**	*Maternal hypertension*	*5.0% (3.1–7.6)*
	5	*Other maternal disorders*	*3.9% (2.4–5.8)*	***Motor vehicle road inj.***	***3.6% (2.9–4.4)***	**Ischemic heart disease**	**3.6% (3–4.4)**
	6	**Ischemic heart disease**	**3.6% (3–4.3)**	**Ischemic heart disease**	3.5% (2.9–4.3)	***Motor vehicle road inj.***	***3.5% (2.9–4.3)***
	7	**Hemorrhagic stroke**	**2.9% (2.4–3.5)**	***Self-harm***	3.3% (2.4–6.2)	***Self-harm***	***3.1% (2.3–6.5)***
	8	***Motor vehicle road inj.***	***2.8% (2.2–3.7)***	**Hemorrhagic stroke**	2.6% (2.2–3.2)	*Other maternal disorders*	*2.9% (1.7–4.5)*
	9	*Diarrheal diseases*	*2.8% (2–3.9)*	*Abortion, miscarriage, ectopic*	*2.3% (1.5–3.3)*	**Hemorrhagic stroke**	**2.6% (2.1–3.3)**
	10	*Abortion, miscarriage, ectopic*	*2.8% (1.7–4.4)*	*Other maternal disorders*	*2.2% (1.6–3.1)*	**Cirrhosis other**	**2.2% (1.5–3)**

	Top ten causes of mortality in females and males						
	Males						
	1990			2005		2015	
	Rank	Cause	Proportion	Cause	Proportion	Cause	Proportion
10–14 years	1	***Natural disaster***	***9.8% (15.1–3.9)***	***Natural disaster***	***16.1% (9.4–22.1)***	***War and legal intervention***	***12.2% (5.5–18.1)***
	2	***Drowning***	***8.6% (10.9–6.5)***	***Drowning***	***6.4% (5.2–7.7)***	***Other unintentional***	***6.9% (4.6–10.1)***
	3	*Lower respiratory infect.*	*5.4% (7.1–3.4)*	***Motor vehicle road inj.***	***5.7% (4.8–7)***	***Drowning***	***6.4% (5.2–7.9)***
	4	***Motor vehicle road inj.***	***5.3% (6.3–4.2)***	***Other unintentional***	***5.6% (3.6–8.2)***	***Motor vehicle road inj.***	***6.0% (4.8–7.5)***
	5	***Pedestrian road inj.***	***5.3% (6.6–4)***	***Pedestrian road inj.***	***5.0% (3.9–6.2)***	*Lower respiratory infect.*	*4.8% (3.5–6.3)*
	6	*Diarrheal diseases*	*4.1% (6–2.7)*	*Lower respiratory infect.*	*4.2% (3.2–5.5)*	***Pedestrian road inj.***	***4.7% (3.6–5.9)***
	7	*Measles*	*3.7% (9.2–1)*	*Typhoid fever*	*4.0% (2.2–6.8)*	*Typhoid fever*	*4.0% (2.1–7)*
	8	***Other unintentional***	***3.7% (5.4–2.5)***	*Diarrheal diseases*	*3.1% (1.9–4.7)*	*Diarrheal diseases*	*2.9% (1.8–4.6)*
	9	*Typhoid fever*	*3.6% (6.1–1.9)*	**Hemorrhagic stroke**	**2.3% (1.9–2.6)**	**Congenital heart**	**2.6% (1.8–3.4)**
	10	**Hemorrhagic stroke**	**3.3% (4.1–2.7)**	**Congenital heart**	**2.2% (1.4–2.9)**	**Hemorrhagic stroke**	**2.2% (1.9–2.6)**
15–19 years	1	***Natural disaster***	***9.6%(3.8–14.8)***	***Natural disaster***	***12.5% (7.2–17.5)***	***War and legal intervention***	***27.2% (13.2–37.9)***
	2	***Motor vehicle road inj.***	***8.9% (6.8–11.8)***	***Motor vehicle road inj.***	***9.8% (8.1–12.1)***	***Other unintentional***	***8.4% (5–12.5)***
	3	***Drowning***	***6.7% (4.8–8.9)***	***Other unintentional***	***7.9% (5.2–11.3)***	***Motor vehicle road inj.***	***8.4% (6.5–10.8)***
	4	***Other unintentional***	***5.3% (3.5–7.5)***	***Pedestrian road inj.***	***5.4% (4.1–6.8)***	***Drowning***	***4.0% (3.1–5.2)***
	5	***Pedestrian road inj.***	***4.7% (2.9–6.2)***	***Drowning***	***5.1% (4.3–6.2)***	***Pedestrian road inj.***	***4.0% (2.9–5.3)***
	6	***War and legal intervention***	***4.3% (1.5–9.3)***	***Motorcyclist road inj.***	***4.0% (2.9–5.4)***	***Self-harm***	***2.9% (2.2–3.7)***
	7	***Self-harm***	***3.1% (2.4–4)***	***Self-harm***	***3.5% (2.8–4.2)***	***Motorcyclist road inj.***	***2.8% (1.8–3.9)***
	8	***Motorcyclist road inj.***	***3.0% (1.8–4.5)***	***War and legal intervention***	***3.4% (1.7–5)***	*Lower respiratory infect.*	*2.3% (1.6–3.1)*
	9	*Lower respiratory infect.*	*3.0% (2–3.8)*	*Lower respiratory infect.*	*2.4% (1.8–3.1)*	***Physical violence by firearm***	***2.2% (1.2–3.3)***
	10	**Hemorrhagic stroke**	**2.8% (2.2–3.4)**	**Hemorrhagic stroke**	**2.2% (1.8–2.5)**	***Other physical violence***	***1.8% (0.8–2.9)***
20–24 years	1	***Motor vehicle road inj.***	***9.9% (7.7–12.9)***	***Motor vehicle road inj.***	***11.1% (9.4–13.4)***	***War and legal intervention***	***27.7% (14.2–38.4)***
	2	***Natural disaster***	***6.8% (2.7–10.8)***	***Natural disaster***	***8.9% (5–12.8)***	***Motor vehicle road inj.***	***8.9% (7–11.3)***
	3	***War and legal intervention***	***5.3% (1.8–11.5)***	***Other unintentional***	***6.8% (4.4–10.1)***	***Other unintentional***	***6.8% (4–10.3)***
	4	***Pedestrian road inj.***	***5.0% (3.1–6.5)***	***Pedestrian road inj.***	***5.7% (4.4–7)***	***Pedestrian road inj.***	***4.2% (3.1–5.4)***
	5	***Other unintentional***	***5.0% (3.3–7.1)***	***Self-harm***	***4.6% (3.8–5.5)***	***Self-harm***	***3.8% (3–5)***
	6	***Drowning***	***5.0% (3.4–6.7)***	***War and legal intervention***	***4.3% (2.2–6.4)***	***Motorcyclist road inj.***	***2.8% (2–4)***
	7	***Self-harm***	***4.2% (3.3–5.4)***	***Motorcyclist road inj.***	***4.1% (3–5.6)***	***Drowning***	***2.8% (2.2–3.6)***
	8	**Tuberculosis**	**3.7% (2.4–5.9)**	***Drowning***	***3.7% (3.1–4.4)***	***Other physical violence***	***2.6% (1–4)***
	9	***Motorcyclist road inj.***	***3.1% (1.8–4.7)***	***Other physical violence***	***3.0% (1.3–4.1)***	***Physical violence by firearm***	***2.4% (1.3–3.6)***
	10	**Ischemic heart disease**	**3.0% (2.5–3.6)**	**Ischemic heart disease**	**2.7% (2.3–3.2)**	**Ischemic heart disease**	**2.3% (1.8–2.9)**

Global burden of disease study 2015, Eastern Mediterranean Region, 1990–2015

*Italic* defines communicable, maternal, neonatal and nutritional diseases

*Bold* defines non-communicable diseases

*Bold, italic* defines injuries

### YLDs

All-cause YLD rates are similar for males and females in the region, and have seen little improvement since 1990 (e-Figure 1 panel B). The leading causes of YLDs are detailed in Table 3. While the burden of some communicable, maternal, and nutritional disorders has declined, this has largely been offset by an increase in disability due to injury among males and lack of reduction in YLDs from non-communicable disease. From 1990 to 2015, iron deficiency anemia was the leading cause of disability for females aged 10–14 and 15–19 years, and for males aged 10–14 years. NCDs, particularly mental health disorders, migraine, asthma, skin conditions, and musculoskeletal disorders, were major contributors to YLDs for both sexes in 2015. Major depression emerged as the leading cause of morbidity amongst males aged 15–19 (7.0%, uncertainty 4.6–9.9) and 20–24 years (8.0%, uncertainty 5.1–11.7) and for females aged 20–24 years (9.1%, uncertainty 6.2–12.4) in 2015. Among older males, opioid use disorders and war were also important causes of disability.

**Table 3:** Leading contributors to poor health (YLDs) for adolescents in the Eastern Mediterranean Region, in 1990, 2005, 2015

	Top ten causes of years lived with a disability (YLDs) in females and males						
	Females						
	1990			2005		2015	
	Rank	Cause	Proportion	Cause	Proportion	Cause	Proportion
10–14 years	1	*Iron-deficiency anemia*	*15.7% (13.5–17.6)*	*Iron-deficiency anemia*	*16.4% (13.9–18.6)*	*Iron-deficiency anemia*	*16.7% (14.3–18.9)*
	2	**Migraine**	**7.0% (4.4–9.9)**	**Migraine**	**7.5% (4.7–10.7)**	**Migraine**	**7.6% (4.8–10.8)**
	3	**Anxiety disorders**	**6.4% (4.6–8.8)**	**Anxiety disorders**	**6.8% (4.9–9.3)**	**Anxiety disorders**	**7.0% (5–9.5)**
	4	**Asthma**	**5.6% (4.2–7.4)**	**Conduct disorder**	**5.8% (3.3–8.6)**	**Conduct disorder**	**5.9% (3.4–8.8)**
	5	**Conduct disorder**	**5.5% (3.1–8.4)**	**Asthma**	**5.3% (3.9–6.9)**	**Asthma**	**5.7% (4.1–7.6)**
	6	**Major depressive disorder**	**4.1% (2.6–6)**	**Major depressive disorder**	**4.3% (2.7–6.2)**	**Major depressive disorder**	**4.4% (2.8–6.3)**
	7	**Low back pain**	**3.0% (2.3–3.9)**	**Acne vulgaris**	**3.1% (1.7–5.3)**	**Acne vulgaris**	**3.2% (1.7–5.5)**
	3	**Epilepsy**	**2.9% (1.9–3.9)**	**Low back pain**	**3.0% (2.3–4)**	**Low hack pain**	**3.0% (2.3–4)**
	9	**Acne vulgaris**	**2.8% (1.5–4.8)**	**Epilepsy**	**2.8% (2–3.8)**	**Dermatitis**	**2.8% (2.1–3.6)**
	10	**Dermatitis**	**2.5% (1.9–3.3)**	**Dermatitis**	**2.7% (2–3.6)**	**Epilepsy**	**2.6% (1.8–3.7)**
15–19 years	1	*Iron-deficiency anemia*	*10.3% (8.2–12.3)*	*Iron-deficiency anemia*	*10.1% (8.3–12)*	*Iron-deficiency anemia*	*10.0% (8.1–11.9)*
	2	**Major depressive disorder**	**8.0% (5.6–10.9)**	**Major depressive disorder**	**8.1% (5.7–11)**	**Migraine**	**8.3% (5.3–11.9)**
	3	**Migraine**	**7.9% (4.9–11.3)**	**Migraine**	**8.1% (5.1– 11.4)**	**Major depressive disorder**	**8.3% (5.9–11.2)**
	4	**Anxiety disorders**	**6.8% (5–9.2)**	**Anxiety disorders**	**7.0% (5.2–9.4)**	**Anxiety disorders**	**7.2% (5.3–9.7)**
	5	**Low back pain**	**5.0% (3.8–6.4)**	**Low back pain**	**5.1% (3.9–6.6)**	**Low hack pain**	**5.0% (3.8–6.5)**
	6	**Acne vulgaris**	**3.5% (1.8–5.9)**	**Acne vulgaris**	**3.7% (2–6.3)**	**Acne vulgaris**	**3.9% (2–6.6)**
	7	**Asthma**	**3.4% (2.4–4.4)**	**Other musculoskeletal**	**3.7% (2.6–5.1)**	**Other musculoskeletal**	**3.9% (2.7–5.5)**
	8	**Conduct disorder**	**3.1% (1.8–4.9)**	**Conduct disorder**	**3.2% (1.9–5)**	**Conduct disorder**	**3.3% (1.9–5.1)**
	9	**Other musculoskeletal**	**3.0% (2.1–4.2)**	**Asthma**	**3.2% (2.3–4.2)**	**Asthma**	**3.3% (2.3–4.3)**
	10	**Epilepsy**	**2.2% (1.5–3)**	**Epilepsy**	**2.1% (1.5–2.8)**	**Epilepsy**	**2.0% (1.4–2.8)**
20–24 years	1	*Iron-deficiency anemia*	*10.2% (8.1–12.3)*	*Iron-deficiency anemia*	*9.0% (7.1–11)*	**Major depressive disorder**	**9.1% (6.2–12.4)**
	2	**Major depressive disorder**	**8.7% (5.9–11.8)**	**Major depressive disorder**	**8.8% (6–12.1)**	*Iron-deficiency anemia*	*8.9% (7–10.8)*
	3	**Migraine**	**7.8% (4.9–11.4)**	**Migraine**	**8.0% (5–11.6)**	**Migraine**	**8.3% (5.3–12)**
	4	**Anxiety disorders**	**6.0% (4.4–8.2)**	**Anxiety disorders**	**6.2% (4.5–8.5)**	**Anxiety disorders**	**6.4% (4.7–8.6)**
	5	**Low back pain**	**6.0% (4.6–7.7)**	**Low back pain**	**6.1%(4.7–7.9)**	**Low back pain**	**6.1% (4.7–7.9)**
	6	**Other musculoskeletal**	**4.2% (3–5.6)**	**Other musculoskeletal**	**4.9% (3.5–6.6)**	**Other musculoskeletal**	**5.4% (3.9–7.3)**
	7	**Bipolar disorder**	**2.4% (1.6–3.5)**	**Neck pain**	**2.5% (1.7–3.4)**	**Bipolar disorder**	**2.5% (1.7–3.7)**
	8	**Neck pain**	**2.3% (1.5–3.2)**	**Bipolar disorder**	**2.4% (1.6–3.5)**	**Neck pain**	**2.4% (1.6–3.3)**
	9	**Other mental and substance**	**2.2% (1.7–2.8)**	**Other mental and substance**	**2.3% (1.8–2.9)**	**Other mental and substance**	**2.4% (1.9–3.1)**
	10	**Premenstrual syndrome**	**1.9% (1.4–2.6)**	**Premenstrual syndrome**	**1.9% (1.4–2.6)**	**Medication overuse**	**1.9% (1.2–3)**

	Top ten causes of years lived with a disability (YLDs) in females and males						
	Males						
	1990			2005		2015	
	Rank	Cause	Proportion	Cause	Proportion	Cause	Proportion
10–14 years	1	*Iron-deficiency anemia*	*18.9% (16.6–20.9)*	*Iron-deficiency anemia*	*19.4% (17–21.5)*	*Iron-deficiency anemia*	*19.5% (17–21.6)*
	2	**Conduct disorder**	**7.5% (4.7–10.7)**	**Conduct disorder**	**7.9% (5–11.3)**	**Conduct disorder**	**8.1% (5.1–11.5)**
	3	**Asthma**	**5.3% (3.9–6.9)**	**Asthma**	**5.2% (3.8–6.8)**	**Asthma**	**5.1% (3.7–6.8)**
	4	**Anxiety disorders**	**4.0% (2.8–5.5)**	**Anxiety disorders**	**4.2% (3–5.9)**	**Anxiety disorders**	**4.3% (3.1–6.1)**
	5	**Migraine**	**3.3% (2–4.8)**	**Migraine**	**3.5% (2.2–5.2)**	**Migraine**	**3.6% (2.2–5.3)**
	6	**Low back pain**	**2.9% (2.2–3.9)**	**Low back pain**	**3.1% (2.3–4)**	**Low back pain**	**3.0% (23–4.1)**
	7	**Epilepsy**	**2.9% (2–3.9)**	**Major depressive disorder**	**2.9% (1.7–4.5)**	**Major depressive disorder**	**3.0% (1.8–4.6)**
	8	**Major depressive disorder**	**2.7% (1.6–4.3)**	**Acne vulgaris**	**2.8% (1.5–4.7)**	**Acne vulgaris**	**2.9% (1.5–4.9)**
	9	**Acne vulgaris**	**2.5% (1.3–4.2)**	**Epilepsy**	**2.6% (1.8–3.4)**	**Epilepsy**	**2.5% (1.7–3.5)**
	10	**Thalassemias trait**	**2.2% (1.9–2.4)**	**Thalassemias trait**	**2.4% (2.1–2.6)**	**Thalassemias trait**	**2.4% (2.1–2.7)**
15–19 years	1	*Iron-deficiency anemia*	*7.7% (6.2–9.6)*	**Major depressive disorder**	**6.9% (4.5–9.9)**	**Major depressive disorder**	**7.0% (4.6–9.9)**
	2	**Major depressive disorder**	**6.6% (4.3–9.6)**	*Iron-deficiency anemia*	*6.8% (5.2–8.6)*	*Iron-deficiency anemia*	*6.5% (5–8.3)*
	3	**Low back pain**	**6.3% (4.9–8.1)**	**Low back pain**	**6.5% (4.9–8.3)**	**Low back pain**	**6.4% (4.8–8.3)**
	4	**Conduct disorder**	**5.7% (3.5–8.3)**	**Conduct disorder**	**6.0% (3.8–8.7)**	**Conduct disorder**	**6.0% (3.7–8.8)**
	5	**Migraine**	**4.9% (3–7.1)**	**Migraine**	**5.2% (3.2–7.5)**	**Migraine**	**5.3% (3.3–7.7)**
	6	**Anxiety disorders**	**4.6% (3.3–6.3)**	**Anxiety disorders**	**4.9% (3.5–6.6)**	**Anxiety disorders**	**4.9% (3.6–6.7)**
	7	**Acne vulgaris**	**3.7% (1.9–6.4)**	**Acne vulgaris**	**4.1% (2.1–6.8)**	**Acne vulgaris**	**4.2% (2.2–7.1)**
	8	**Asthma**	**3.5% (2.6–4.6)**	**Other musculoskeletal**	**3.8% (2.7–5.3)**	**Other musculoskeletal**	**4.0% (2.7–5.6)**
	9	**Other musculoskeletal**	**3.0% (2–4.3)**	**Asthma**	**3.5% (2.5–4.6)**	**Asthma**	**3.3% (2.4–4.5)**
	10	**Epilepsy**	**2.6% (1.9–3.5)**	**Epilepsy**	**2.5% (1.8–3.3)**	***War and legal intervention***	***2.7% (1.2–5)***
20–24 years	1	**Major depressive disorder**	**7.7% (4.9–11.4)**	**Major depressive disorder**	**7.9% (5–11.8)**	**Major depressive disorder**	**8.0% (5.1–11.7)**
	2	**Low back pain**	**7.3% (5.7–9.4)**	**Low back pain**	**7.4% (5.7–9.4)**	**Low back pain**	**7.4% (5.7–9.4)**
	3	**Migraine**	**5.7% (3.7–8.1)**	**Migraine**	**5.8% (3.8–8.3)**	**Migraine**	**5.9% (3.8–8.5)**
	4	**Other mental and substance**	**4.2% (3.4–5.3)**	**Opioid use disorders**	**5.1% (3.6–6.8)**	**Other musculoskeletal**	**4.9% (3.5–6.6)**
	5	*Iron-deficiency anemia*	*4.2% (3–5.5)*	**Other musculoskeletal**	**4.6% (3.3–6.1)**	**Opioid use disorders**	**4.9% (3.5–6.6)**
	6	**Opioid use disorders**	**4.2% (2.9–5.6)**	**Other mental and substance**	**4.4% (3.6–5.5)**	**Other mental and substance**	**4.5% (3.5–5.5)**
	7	**Anxiety disorders**	**3.9% (2.7–5.5)**	**Anxiety disorders**	**4.0% (2.8–5.5)**	**Anxiety disorders**	**4.0% (2.8–5.7)**
	8	***War and legal intervention***	***3.8% (2–6.1)***	*Iron-deficiency anemia*	*3.6% (2.6–4.8)*	***War and legal intervention***	***3.6% (1.6–6.3)***
	9	**Other musculoskeletal**	**3.5% (2.5–4.8)**	**Neck pain**	**2.5% (1.7–3.5)**	*Iron-deficiency anemia*	*3.1% (2.2–4.1)*
	10	***Other unintentional inj.***	***2.3% (2–2.7)***	**Bipolar disorder**	**2.4% (1.6–3.5)**	**Bipolar disorder**	**2.4% (1.6–3.5)**

Global burden of disease study 2015, Eastern Mediterranean Region, 1990–2015

*Italic* defines communicable, maternal, neonatal and nutritional diseases

*Bold* defines non-communicable diseases

*Bold, italic* defines injuries

*YLDs* years of life with disability

### DALYs

All-cause DALY rates have declined for females of all ages and 10- to 14-year-old males in the region (e-Figure 1, panel c), largely due to a reduction of communicable, maternal, and nutritional diseases. DALY rates have increased for males aged 15–19 and 20–24 since 1990, largely due to an increased burden of injury. The leading causes of DALYs by age and sex are provided in Table 4. For females, type 1 conditions (nutritional disorders, communicable disease, and maternal disorders) remain important causes of DALYs. However, non-communicable diseases (mental health disorders, migraine, skin conditions, and musculoskeletal disorders) account for more than half of the total disease burden. For males, injuries due to conflict, transport, and other unintentional injuries are the leading causes of DALYs, particularly amongst 15- to 24-year olds.

**Table 4:** Leading contributors to poor health (DALYs) for adolescents in the Eastern Mediterranean Region, in 1990, 2005, 2015

	Top ten causes of disability- adjusted life years (DALYs) in females and males						
	Females						
	1990			2005		2015	
	Rank	Cause	Proportion	Cause	Proportion	Cause	Proportion
10–14 years	1	*Iron-deficiency anemia*	*7.8% (6.1–9.8)*	*Iron-deficiency anemia*	*8.8% (6.9–11)*	*Iron-deficiency anemia*	*9.9% (7.9–12.1)*
	2	***Natural disaster***	***4.5% (2–6.9)***	***Natural disaster***	***7.0% (4–9.8)***	***War and legal intervention***	***4.5% (2.3–6.9)***
	3	*Diarrheal diseases*	*3.4% (2.5–4.6)*	**Migraine**	**4.0% (2.5–5.7)**	**Migraine**	**4.5% (2.8–6.4)**
	4	**Migraine**	**3.4% (2.1–5)**	**Anxiety disorders**	**3.6% (2.5–4.9)**	**Anxiety disorders**	**4.1% (2.9–5.6)**
	5	**Asthma**	**3.3% (2.4–4.3)**	**Asthma**	**3.3% (2.5–4.3)**	**Asthma**	**3.8% (2.7–5)**
	6	**Anxiety disorders**	**3.2% (2.2–4.3)**	**Conduct disorder**	**3.1% (1.8–4.7)**	**Conduct disorder**	**3.5% (2–5.3)**
	7	*Lower respiratory infect.*	*3.0% (1.8–4)*	*Diarrheal diseases*	*2.7% 12–3 .8)*	**Major depressive disorder**	**2.6 % (1.6–3.7)**
	8	*Measles*	*2.7% (0.8–6.5)*	*Typhoid fever*	*2.5% (1.4–4.2)*	*Lower respiratory infect.*	*2.3% (1.5–3.2)*
	9	**Conduct disorder**	**2.7% (1.5–4.2)**	**Major depressive disorder**	**2.3% (1.4–3.3)**	**Epilepsy**	**2.2% (1.6–2.9)**
	10	*Tuberculosis*	*2.4% (1.3–3.7)*	*Lower respiratory infect.*	*2.2% (1.5–3)*	*Diarrheal diseases*	*2.2% (1.6–3.1)*
15–19 years	1	*Iron-deficiency anemia*	*5.3% (3.9–6.8)*	*Iron-deficiency anemia*	*5.4% (4.1–6.8)*	*Iron-deficiency anemia*	*5.5% (4.2–7.1)*
	2	**Major depressive disorder**	**4.0% (2.7–5.6)**	***Natural disaster***	***4.6% (2.7–6.4)***	***War and legal intervention***	***5.3% (2.6–8.1)***
	3	**Migraine**	**4.0% (2.4–5.7)**	**Major depressive disorder**	**4.2% (2.9–5.9)**	**Migraine**	**4.6% (2.8–6.6)**
	4	**Anxiety disorders**	**3.4% (2.4–4.7)**	**Migraine**	**4.2% (2.6–6.1)**	**Major depressive disorder**	**4.5% (3.1–6.3)**
	5	***Natural disaster***	***3.2% (1.4–5.1)***	**Anxiety disorders**	**3.7% (2.6–4.9)**	**Anxiety disorders**	**3.9% (2.8–5.3)**
	6	*Tuberculosis*	*3.1% (1.9–4.7)*	**Low back pain**	**2.6% (2–3.5)**	**Low back pain**	**2.7% (2–3.6)**
	7	*Maternal hemorrhage*	*2.7% (1.9–3.9)*	*Tuberculosis*	*2.6% (1.6–3.7)*	*Tuberculosis*	*2.3% (1.4–3.3)*
	8	**Low back pain**	**2.5% (1.9–3.3)**	*Maternal hemorrhage*	*2.6% (1.8–3.7)*	**Other musculoskeletal**	**2.3% (1.6–3.1)**
	9	*Diarrheal diseases*	*2.4% (1.8–3.3)*	**Asthma**	**2.1% (1.5–2.7)**	**Asthma**	**2.2% (1.6–2.9)**
	10	**Asthma**	**2.2% (1.7–2.8)**	**Other musculoskeletal**	**2.0% (1.4–2.8)**	**Acne vulgaris**	**2.2% (1.1–3.8)**
20–24 years	1	*Iron-deficiency anemia*	*5.0% (3.7–6.6)*	*Iron-deficiency anemia*	*4.7% (3.5–6.2)*	**Major depressive disorder**	**5.0% (3.3–7)**
	2	*Maternal hemorrhage*	*4.2% (3–5.8)*	**Major depressive disorder**	**4.6% (3–6.5)**	*Iron-deficiency anemia*	*4.9% (3.6–6.3)*
	3	**Major depressive disorder**	**4.2% (2.8–6)**	**Migraine**	**4.1% (2.6–6.1)**	**Migraine**	**4.5% (2.9–6.7)**
	4	*Tuberculosis*	*3.9% (2.5–5.6)*	*Maternal hemorrhage*	*3.7% (2.6–5.1)*	***War and legal intervention***	***3.7% (1.8–5.6)***
	5	**Migraine**	**3.8% (2.4–5.6)**	***Natural disaster***	***3.5% (2.1–5)***	**Anxiety disorders**	**3.5% (2.4–4.8)**
	6	*Maternal hypertension*	*3.1% (2–4.4)*	**Anxiety disorders**	**3.2% (2.3–4.4)**	**Low back pain**	**3.3% (2.4–4.5)**
	7	**Anxiety disorders**	**2.9% (2–4)**	*Tuberculosis*	*3.2% (2.1–4.5)*	**Other musculoskeletal**	**3.1% (2.2–4.2)**
	8	**Low back pain**	**2.9% (2.1–3.8)**	**Low back pain**	**3.2% (2.3–4.2)**	*Tuberculosis*	*2.9% (1.9–4)*
	9	***Natural disaster***	***2.3% (0.9–3.7)***	*Maternal hypertension*	*2.7% (1.9–3.8)*	*Maternal hemorrhage*	*2.6% (1.6–3.8)*
	10	*Other maternal disorders*	*2.1% (1.3–3.1)*	**Other musculoskeletal**	**2.7% (1.9–3.6)**	*Maternal hypertension*	*2.4% (1.5–3.7)*

	Top ten causes of disability- adjusted life years (DALYs) in females and males						
	Males						
	1990			2005		2015	
	Rank	Cause	Proportion	Cause	Proportion	Cause	Proportion
10–14 years	1	*Iron-deficiency anemia*	*8.4% (6.5–10.6)*	*Iron-deficiency anemia*	*9.5% (7.4–11.8)*	*Iron-deficiency anemia*	*10.6% (8.4–13)*
	2	***Natural disaster***	***5.8% (2.4–9.1)***	***Natural disaster***	***8.9% (5–12.7)***	***War and legal intervention***	***6.2% (2.9–9.5)***
	3	***Drowning***	***4.9% (3.4–6.3)***	**Conduct disorder**	**3.9% (2.4–5.8)**	**Conduct disorder**	4.4% (2.7–6.5)
	4	**Conduct disorder**	**3.3% (2–5)**	***Other unintentional***	***3.6% (2.6–5)***	***Other unintentional***	***4.0% (2.8–5.6)***
	5	***Motor vehicle road inj.***	***3.0% (2.3–4)***	***Drowning***	***3.3% (2.6–4.1)***	**Asthma**	**3.3% (2.5–4.4)**
	6	*Lower respiratory infect.*	*3.0% (2–4.1)*	**Asthma**	**3.2% (2.4–4.1)**	***Drowning***	***2.9% (2.3–3.8)***
	7	**Asthma**	**3.0% (2.3–3.9)**	***Motor vehicle road inj.***	***3.0% (2.4–3.7)***	***Motor vehicle road inj.***	***2.8% (2.2–3.5)***
	8	***Pedestrian road inj.***	***3.0% (2.2–3.9)***	***Pedestrian road inj.***	***2.6% (2–3.2)***	**Anxiety disorders**	**2.3% (1.6–3 2)**
	9	***Other unintentional***	***2.8% (2.2–3.8)***	*Lower respiratory infect.*	*2.2% (1.6–3)*	*Lower respiratory infect.*	*2.3% (1.6–3.1)*
	10	*Diarrheal diseases*	*2.6% (1.8–3.7)*	*Typhoid fever*	*2.1% (1.1–3.6)*	***Pedestrian road inj.***	***2.1% (1.6–2.8)***
15–19 years	1	***Natural disaster***	***6.0 % (2.5–9.3)***	***Natural disaster***	***7.9% (4.4–11.3)***	***War and legal intervention***	***17.9% (8.9–25.7)***
	2	***Motor vehicle road inj.***	***5.3% (4–7.1)***	***Motor vehicle road inj.***	***5.9% (4.8–7.5)***	***Other unintentional***	***5.9% (3.9–8.3)***
	3	***Other unintentional***	***4.0% (3–5.4)***	***Other unintentional***	***5.6% (3.9–7.7)***	***Motor vehicle road inj.***	***5.2% (4.1–6.6)***
	4	***Drowning***	***4.0% (2.8–5.4)***	***Pedestrian road inj.***	***3.2% (2.4–4.1)***	**Major depressive disorder**	**2.7% (1.7–4.1)**
	5	*Iron-deficiency anemia*	*3.3% (2.4–4.4)*	***Drowning***	***3.1% (2.5–3.8)***	*Iron-deficiency anemia*	*2.5% (1.7–3.5)*
	6	***War and legal intervention***	***3.2% (1.5–6.2)***	*Iron-deficiency anemia*	*2.8% (2–3.8)*	***Drowning***	***2.5% (1.9–3.2)***
	7	***Pedestrian road inj.***	***2.8% (1.7–3.7)***	**Major depressive disorder**	**2.8% (1.7–4.2)**	***Pedestrian road inj.***	***2.5% (1.8–3.3)***
	8	**Major depressive disorder**	**2.7% (1.7–4.1)**	**Low back pain**	**2.6% (1.9–3.5)**	**Low back pain**	**2.4% (1.7–3.3)**
	9	**Low back pain**	**2.6% (1.9–3.5)**	***Motorcyclist road inj.***	***2.4% (1.7–3.3)***	**Conduct disorder**	**2.3% (1.4–3.6)**
	10	**Conduct disorder**	**2.3% (1.4–3.6)**	**Conduct disorder**	**2.4% (1.4–3.7)**	**Migraine**	**2.0% (1.2–3)**
20–24 years	1	***Moter vehicle road inj.***	***6.2% (4.9–8.1)***	***Motor vehicle road inj.***	***7.1% (5.8–8.7)***	***War and legal intervention***	***19.5% (10–27.7)***
	2	***War and legal intervention***	***4.7% (2.3–8.7)***	***Natural disaster***	***5.9% (3.3–8.5)***	***Motor vehicle road inj.***	***5.9% (4.7–7.4)***
	3	***Natural disaster***	***4.4% (1.8–7)***	***Other unintentional***	***5.2% (3.7–7.3)***	***Other unintentional***	***5.2% (3.4–7.4)***
	4	***Other unintentional***	***4.0% (2.9–5.4)***	***Pedestrian road inj.***	***3.6% (2.7–4.5)***	***Pedestrian road inj.***	***2.8% (2.1–3.5)***
	5	***Pedestrian road inj.***	***3.1% (1.9–4.2)***	***War and legal intervention***	***3.4% (2–4.8)***	***Major depressive disorder***	***2.7% (1.7–4.3)***
	6	***Drowning***	***3.1% (2.1–4.2)***	**Major depressive disorder**	**2.9% (1.8–4.7)**	***Low back pain***	***2.5% (1.8–3.5)***
	7	**Major depressive disorder**	**2.9% (1.8–4.5)**	***Self-harm***	***2.9% (2.4–3.5)***	***Self-harm***	***2.5% (2–3.2)***
	8	**Low back pain**	**2.8% (2–3.8)**	**Opioid use disorders**	**2.8% (2.2–3.4)**	**Opioid use disorders**	**2.5% (1.9–3.2)**
	9	***Self-harm***	***2.6% (2.1–3.4)***	**Low back pain**	**2.7% (1.9–3.7)**	**Migraine**	**2.0% (1.3–3.1)**
	10	*Tuberculosis*	*2.5% (1.6–3.9)*	***Motorcyclist road inj.***	***2.6% (1.9–3.6)***	***Motorcyclist road inj.***	***1.9% (1.3-2.6)***

Global burden of disease study 2015, Eastern Mediterranean Region, 1990–2015

*Italic* defines communicable, maternal, neonatal and nutritional diseases

*Bold* defines non-communicable diseases

*Bold, italic* defines injuries

*DALYs* disability-adjusted life-years

There was considerable variation in all-cause and cause-specific DALY rates across countries in the region (e-Figure 2, panels A–C). In all countries in the region, DALY rates are highest among males, and higher among 20- to 24-year olds than other ages in males and females. The highest DALY rates were in countries most affected by recent conflict or insecurity and/or those with the lowest SDI, such as Pakistan (17,483 per 100,000 10–24 years in 2015), Somalia (27,716 DALYs per 100,000 10- to 24-year olds in 2015), Afghanistan (32,068 per 100,000 10- to 24-year olds in 2015) and Syria (33,452 per 100,000 10- to 24-year olds in 2015), with 10- to 24-year olds globally having a DALY rate of 14,557 per 100,000 in 2015. In these countries, injury, particularly due to war and legal intervention was a major contributor to DALYs, particularly amongst males. For example, in Syria over 70% of DALYs to 10–24 year olds were due to injury, with males aged 20–24 experiencing the largest burden. A number of these countries also experience a high burden of nutritional disorders and communicable disease among younger adolescents, in addition to a substantial burden of maternal health problems. Countries with a higher SDI and those less affected by conflict experienced a lower burden of poor health. In these settings DALYs were mostly due to NCDs including mental health disorders, skin conditions, asthma, migraine, and musculoskeletal disorders.

The three countries that had the largest populations of adolescents in the EMR (Pakistan, Iran, and Egypt) had very different disease burdens. Egypt (12,418 DALYs per 100,000 10- to 24-year olds in 2015) and Iran (12,624 DALYs per 100,000 10- to 24-year olds in 2015) has similar low rates of DALYs; however, Iran had a higher burden due to injury (3361 per 100,000 in Iran compared to 1938 per 100,000 in Egypt). Pakistan had a high burden of injury (3367 per 100,000) but more so type I conditions (5252 per 100,000).

e-Figure 2, panels D–F shows the expected DALYs in each country based on the SDI. The most striking finding is that with the reduction of DALYs due to war (which is not expected based on development), there remains a very large burden of poor health for adolescents in the EMR.

### Health risks and determinants

The prevalence of selected health risks is provided in e-Figure 3. The prevalence of overweight and obesity was highest among countries with a higher SDI in the region, and was generally similar for males and females. Rates of daily tobacco smoking among males aged 10–24 years ranged from 1.9% in Sudan to 18% in Kuwait, but were less than 5% for females in the region. Similarly, the prevalence of binge drinking was higher among males than females at all ages, but was less than 10% for both sexes in most EMR countries. Unmet need for contraception was high among the 11 countries for which data are available (e-Figure 4). More than one third of females who are married or in union have unmet need for contraception in Pakistan, Djibouti, Somalia, Sudan, and Yemen. These countries also have among the highest rates of adolescent birth rates in the region, adolescent fertility the greatest in Somalia (114.7 live births per 1000 females aged 15–19 in 2015). There was also great variation in educational attainment in the EMR region. Low-SDI countries affected by protracted insecurity and conflict have the lowest mean number of years of completed education, most notably for females. Rates of unemployment among 15- to 24-year olds also vary considerably, and were generally higher for females than males.

## Discussion

This study is the first systematic analysis of adolescent health in the EMR. The findings suggest dramatic shifts in the health of adolescents living in the EMR over the past 25 years. Communicable diseases and the health consequences of natural disasters have reduced substantially, but these gains have largely been offset by war and the emergence of NCDs including mental health disorders, unintentional injury, and self-harm. Indeed, adolescents living in Syria, Afghanistan, and Somalia experience amongst the largest burdens of disease and injury of all adolescents globally (Patton et al. 2016). Even with the return of peace and security to this region, adolescents will have a persisting poor health profile that will pose a barrier to socioeconomic growth and development of the EMR (The World Bank 2006).

The substantial reductions in mortality and morbidity due to communicable disease, maternal disorders, and natural disasters in the EMR are likely the result of socioeconomic growth and development, educational participation, and interventions through the health system. Recent wars and civil conflict, however, threaten the foundations on which these gains were made, with the risk of resurgence of many of these conditions. This is in addition to the growing burden of NCDs and injuries. Renewed efforts to consolidate gains made in communicable disease and maternal health will be required. The Lancet Commission described the benefits that can accrue from investments in adolescence. Without these investments, the EMR region risks poor health across adolescence, adulthood, and in the next generation (Patton et al. 2016).

War and legal interventions (deaths due to law enforcement, regardless of their legality) were the leading causes of mortality for adolescents aged 10–24 years of both genders in the EMR. While not all countries in the EMR are affected by conflict, the magnitude of mortality in those countries that are affected signifies this as a priority for the region. It should also be noted, however, that at a population level (all ages combined), war and legal intervention is only the fifth leading cause of mortality in the EMR (GBD 2015 Eastern Mediterranean Region Collaborators and Mokdad 2017). This may reflect competing causes of mortality at other ages, or it may signify that war and legal interventions disproportionately affect adolescents, particularly 20- to 24-year-old males. In addition to the physical injuries and disability that accompany conflict, violence and trauma at this critical developmental stage carry a risk of persisting effects on future mental health and well-being. War and conflict are also likely to result in disruption to quality education, social infrastructure, and community development, which have profound implications for health and well-being across the course of one’s life. This may explain why expected DALYs in countries which have experienced large burdens of conflict remain high.

In addition to the health status of adolescents, we explored the burden of risk behavior for future health. Tobacco smoking and high body mass were common risks where interventions during adolescence have the potential to avert later ischemic heart disease, the leading cause of poor health in the region. Of note, estimates of tobacco smoking reported here do not include Sisha smoking which is prevalent in some countries of the EMR and harmful (Maziak et al. 2004). Low rates of education completion in many countries including Afghanistan, Somalia, and Yemen are a major obstacle to growth and future prosperity of this region. High rates of adolescent pregnancy represent an important target for action to improve health and life opportunities for girls and young women. Unmet need for contraception is very high, and is likely to also signify high rates of unmet need for other essential health interventions, particularly for culturally sensitive needs such as sexually transmitted infections and mental health. Given adolescents face barriers in accessing health facilities, there is a need to explore other approaches such as community-based delivery or school health services (Tylee et al. 2007). There is also the need for community-based interventions to address some of the sociocultural barriers that contribute to high unmet need for interventions relating to sexual and reproductive health (Patton et al. 2016).

As over half of the region’s adolescents live in Pakistan, Iran, and Egypt (Table 1), a regional response must prioritize these three countries. This is challenging, however, given the different stages of development and the very different needs of adolescents across these countries. For example, the burden of disease experienced by adolescents in Egypt is predominantly caused by NCDs, which require interventions to address the burden of chronic illness and health risks such as tobacco smoking and obesity (Patton et al. 2016). In addition to NCDs, adolescents living in Iran are burdened by injury (particularly relating to transport injury) which requires a suite of inter-sectoral actions (WHO 2017a). Adolescents living in Pakistan experience a “multi-burden” profile of disease, with a large burden of communicable, nutritional, and reproductive poor health in addition to NCDs and injury. However, in countries such as Afghanistan, Somalia, and Sudan, adolescents represent almost a third of the country-level population and experience a particularly large and complex burden of disease. It is, therefore, important not to neglect their health needs (or indeed other countries in the EMR).

What this analysis highlights is that unintentional injury, mental health, sexual health, substance use, and self-harm are increasingly important health issues for adolescents in the EMR. While these are health issues common to adolescents globally (Patton et al. 2016), they have typically sat at the margins (if at all) of policy, program, and data collection in EMR, given religious and cultural sensitivities. These findings highlight the need to better align health actions, including data monitoring of sensitive health outcomes, including risks, in this region to these needs.

Our analysis has important limitations. Firstly, there is considerable variation in the availability and quality of primary data for adolescent health. This includes paucity of data for some age groups (particularly 10- to 14-year olds), and for many health outcomes and risks of importance during these important developmental years (Mokdad et al. 2016b; Patton et al. 2012; The Global Burden of Disease Child and Adoelscent Health Collaboration 2017). Availability of timely, quality data is likely to be particularly poor in settings of conflict and insecurity, which affects many countries in this region. The poor quality of primary data necessitated the use of modeled estimates, and some of these modeled estimates may have over- or under-estimated the true burden. For example, ischemic heart disease was found to be a leading cause of mortality amongst males aged 20–24 years, a cause of death more commonly associated with adulthood. Premature death due to cardiovascular disease is possible during adolescence, particularly in the context of adolescent obesity which is prevalent in EMR (Franks et al. 2010). This finding may also be an artifact of disease modeling, as ischemic heart disease is the leading cause of mortality in the EMR, and these deaths are modeled to have their onset after 0.1 years of age (GBD 2015 Eastern Mediterranean Region Cardiovascular Disease Collaborators and Mokda 2017; GBD 2015 Eastern Mediterranean Region Collaborators and Mokdad 2017; GBD Mortality and Causes of Death Collaborators 2016). The findings of this study should therefore be interpreted as not only indicating priority areas to address adolescent health in the EMR, but also where data collection efforts should focus. A further limitation is that the broader impacts of armed conflict on adolescent health and well-being, beyond mortality, are not captured by the 2015 GBD study. These include participation in education and employment, as well as the impacts of trauma on adolescent development and wellbeing. Additionally, some important health issues such as female genital cutting/mutilation (common in countries such as Somalia) are not included in GBD 2015 (UNICEF 2016a).

There are several regional efforts that may facilitate addressing the needs of adolescents in the EMR. For example, a coalition of youth advocates for health in the EMR has been established (Alaovie et al. 2017). There is a joint UN strategy for youth in the region (IATTTYP 2015). UNICEF has also published guidance around good practice with adolescent and youth programming (UNICEF 2016b). This study compliments these efforts, and helps to inform some priority areas for health. For conflict-affected countries, the focus must clearly be on the return of peace and stability and the rebuilding of health, education, and social systems. In doing so, it is important to design services that meet the needs of adolescents. For countries not affected by conflict, health actions include the need to re-orientate health systems to focus on prevention and the growing burden of NCDs. This needs to extend to inter-sectoral actions to address the broader determinants of NCDs and injuries. Without urgent action, there is a risk that profiles of adolescent health will continue to deteriorate with consequences for future population health and wellbeing, productivity, and ultimately the stability of civil society.

## Electronic supplementary material

Below is the link to the electronic supplementary material.

Supplementary material 1 (DOCX 4205 kb)

Supplementary material 2 (XLSX 29 kb)
