# Supplementary material for: Adolescent health in the Eastern Mediterranean Region: findings from the global burden of disease 2015 study
Source: Int J Public Health. 2017 Aug 3;63(Suppl 1):79–96. doi: 10.1007/s00038-017-1003-4 (PMC5701730; doi:10.1007/s00038-017-1003-4)
Supplement: Supplementary file 1 — Supplementary material 1 (DOCX 4205 kb) [file 38_2017_1003_MOESM1_ESM.docx]

Electronic Supplementary Material

**Article title:**

Adolescent health in the Eastern Mediterranean Region: Findings from the Global Burden of Disease 2015 Study

**Journal:**

International Journal of Public Health

**Authors:**

GBD 2015 Eastern Mediterranean Region Adolescent Health Collaborators

**Corresponding author:**

Ali H. Mokdad

Institute for Health Metrics and Evaluation, University of Washington, Seattle, WA, United States

Email: [mokdaa@uw.edu](mailto:mokdaa@uw.edu)

**e-Figure 1 - All-cause rates of poor health (mortality, years lost due to disability(YLD) and disability-adjusted life-years (DALYs)) for adolescents in the Eastern Mediterranean Region, 1990-2015. (Global Burden of Disease Study 2015, Eastern Mediterranean Region, 1990-2015).**

Panel A - All-cause mortality rate (per 100,000) and 95% uncertainty for males and females in the Eastern Mediterranean Region.


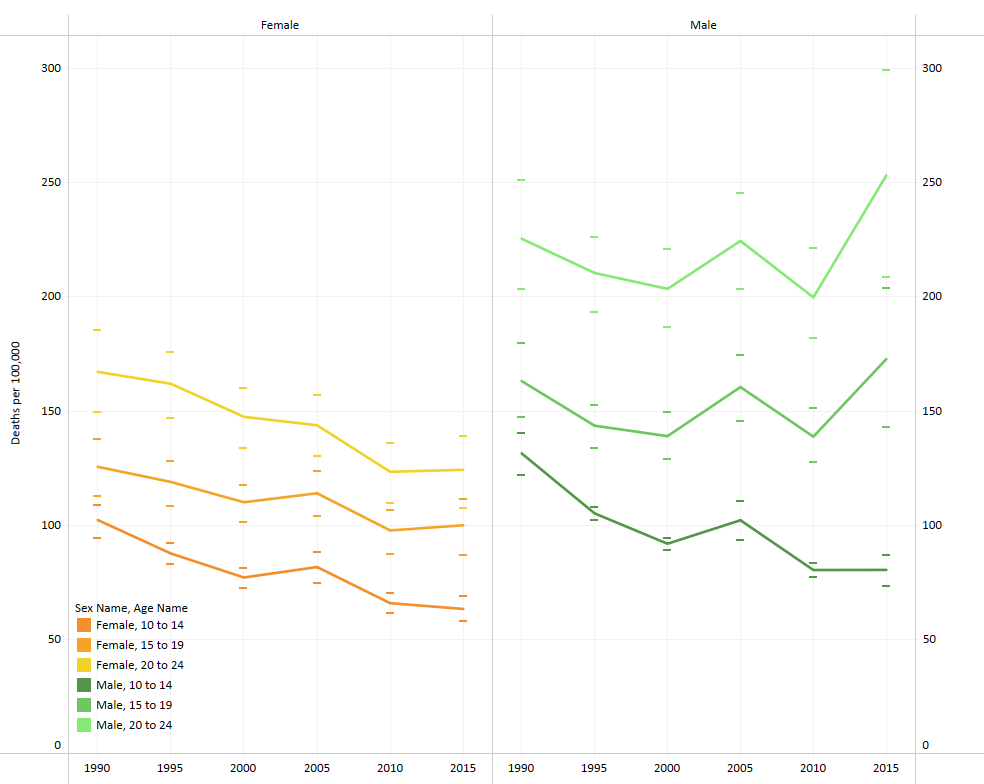


Panel B - All-cause YLDs rate (per 100,000) and 95% uncertainty in the Eastern Mediterranean Region, by sex, 1990-2015.


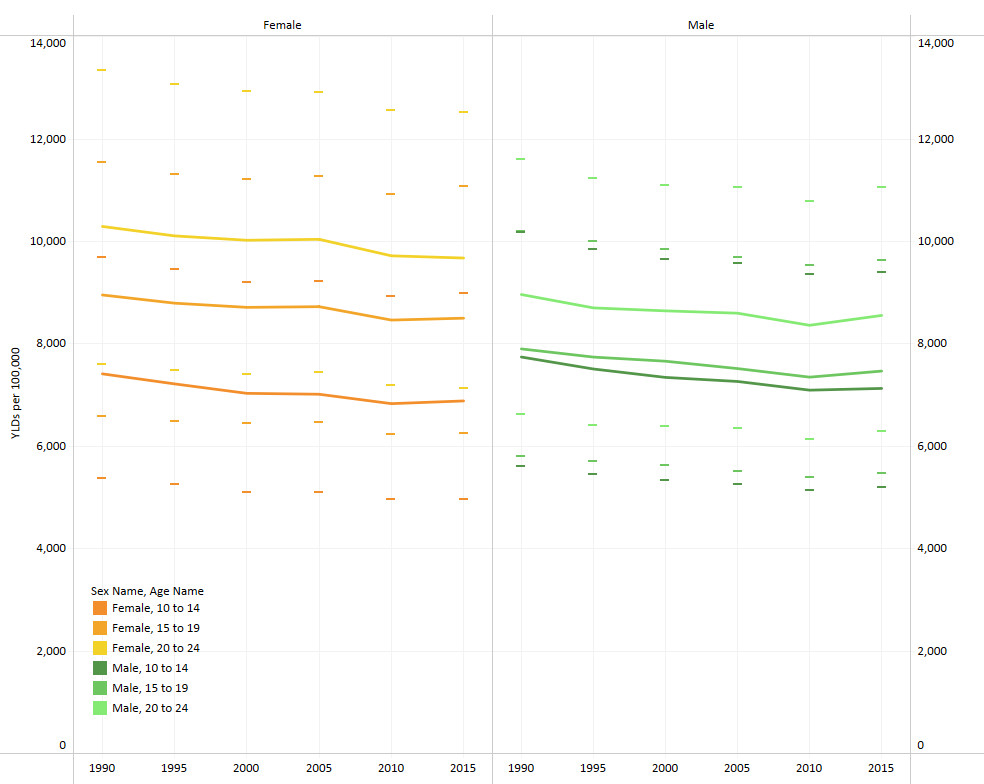


Panel C - All-cause DALYs rate (per 100,000) and 95% uncertainty in the Eastern Mediterranean Region, by sex, 1990-2015.


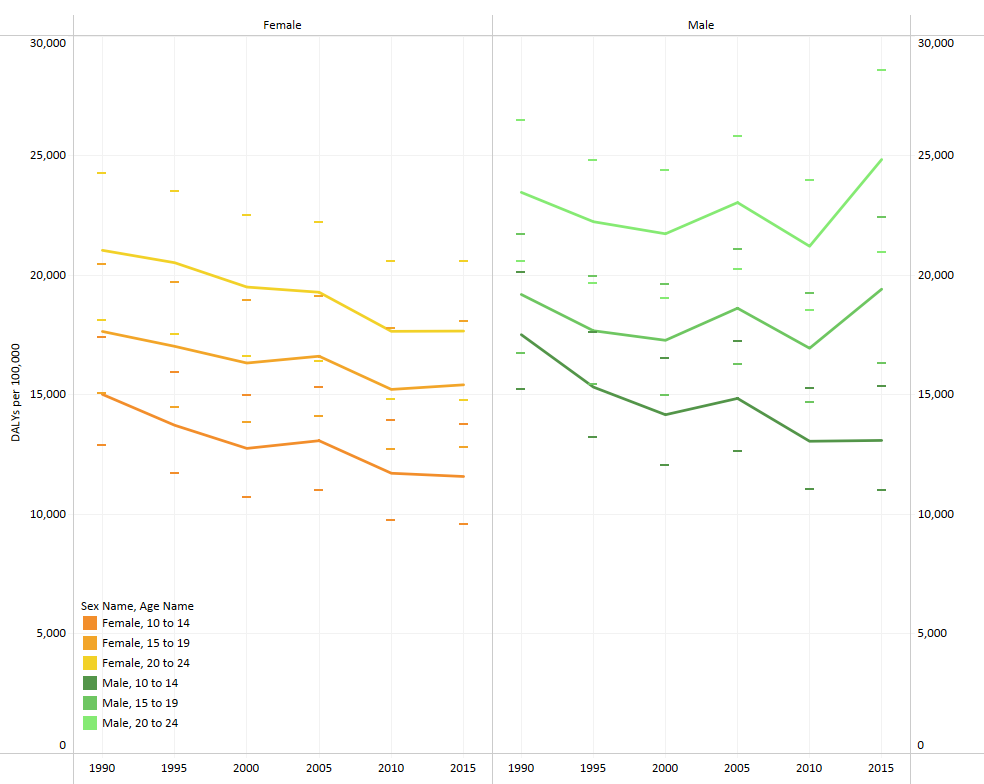


**e-Figure 2 - Observed & expected disability-adjusted life-years(DALYs) for countries in the Eastern Mediterranean Region, by age and gender, in 2015. DALYs = Disability-adjusted life-years. (Global Burden of Disease Study 2015, Eastern Mediterranean Countries, 2015).**

Panel A to C - Observed cause-specific DALYs for countries in the Eastern Mediterranean Region by age group and gender.


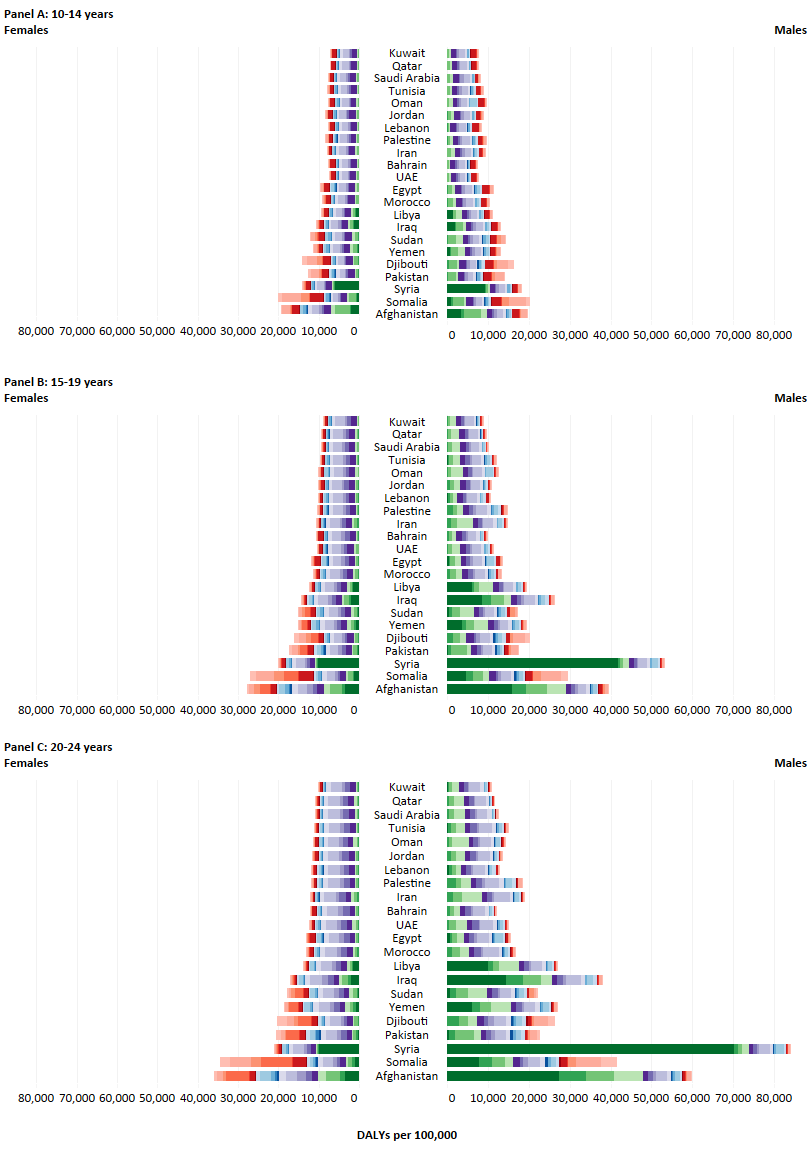


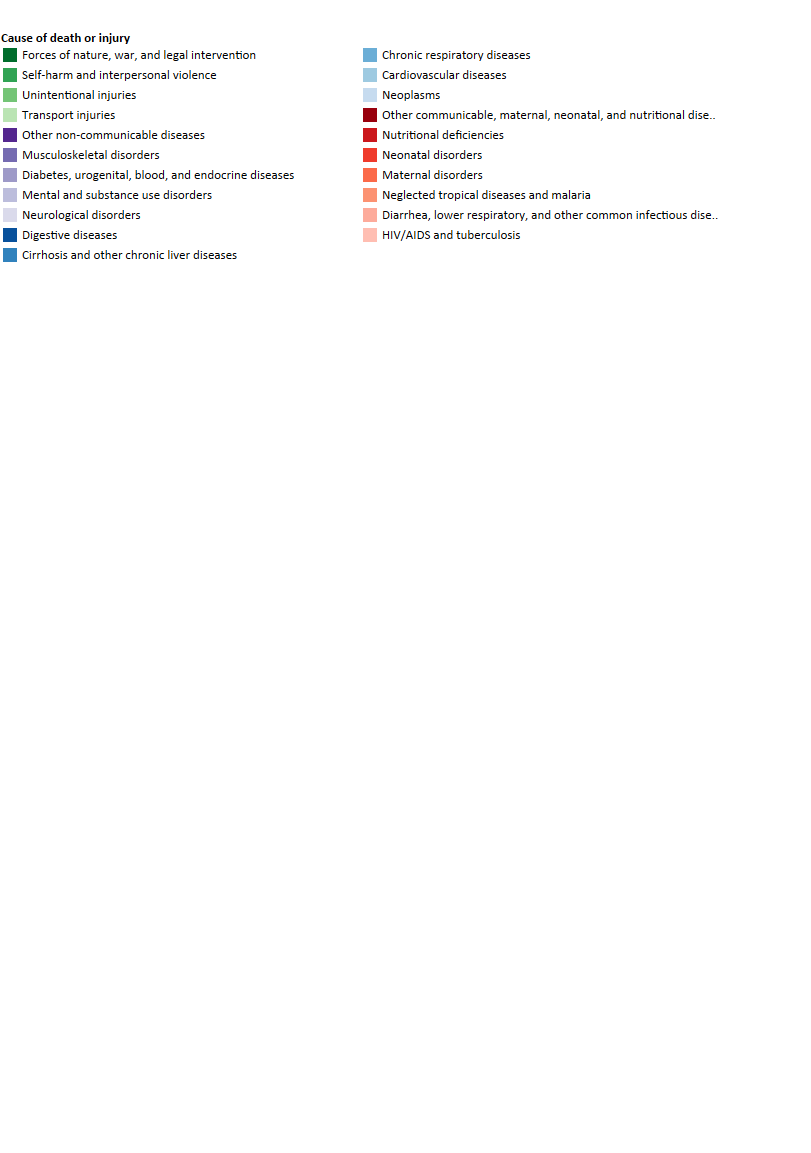


Panel D to F - Expected cause-specific DALYs for countries in the Eastern Mediterranean by age group and gender.


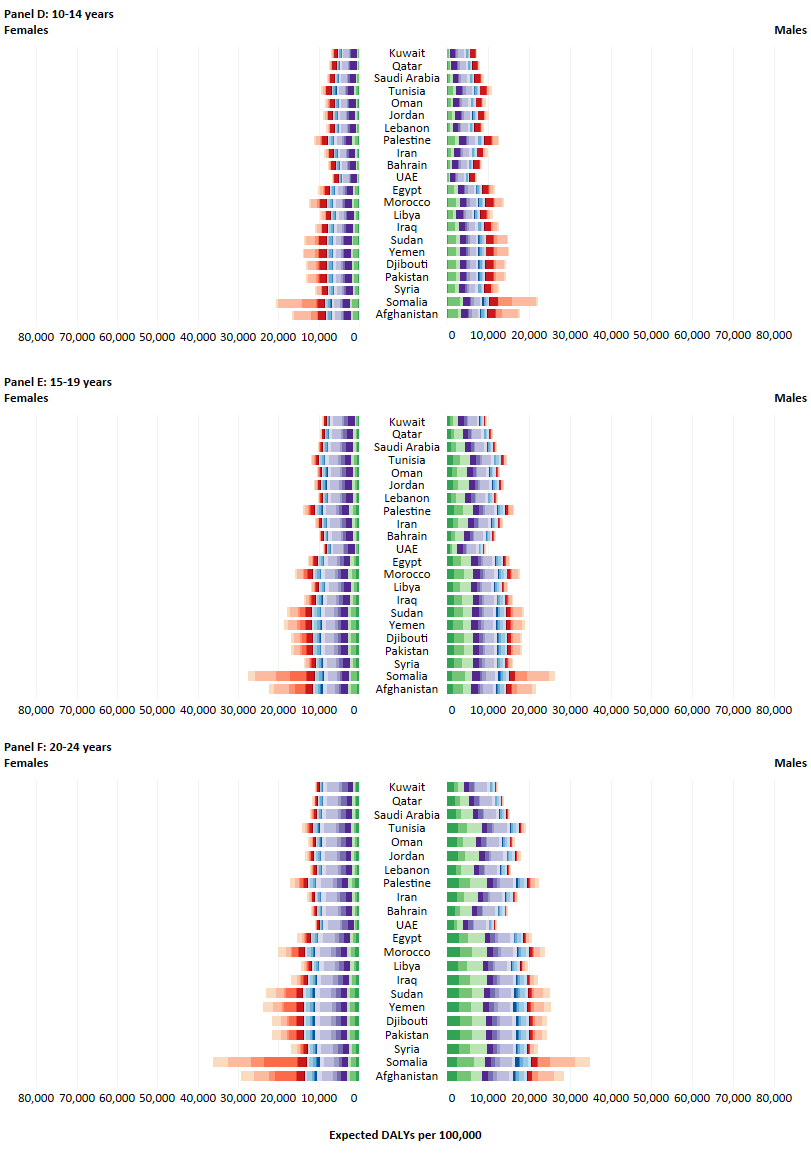


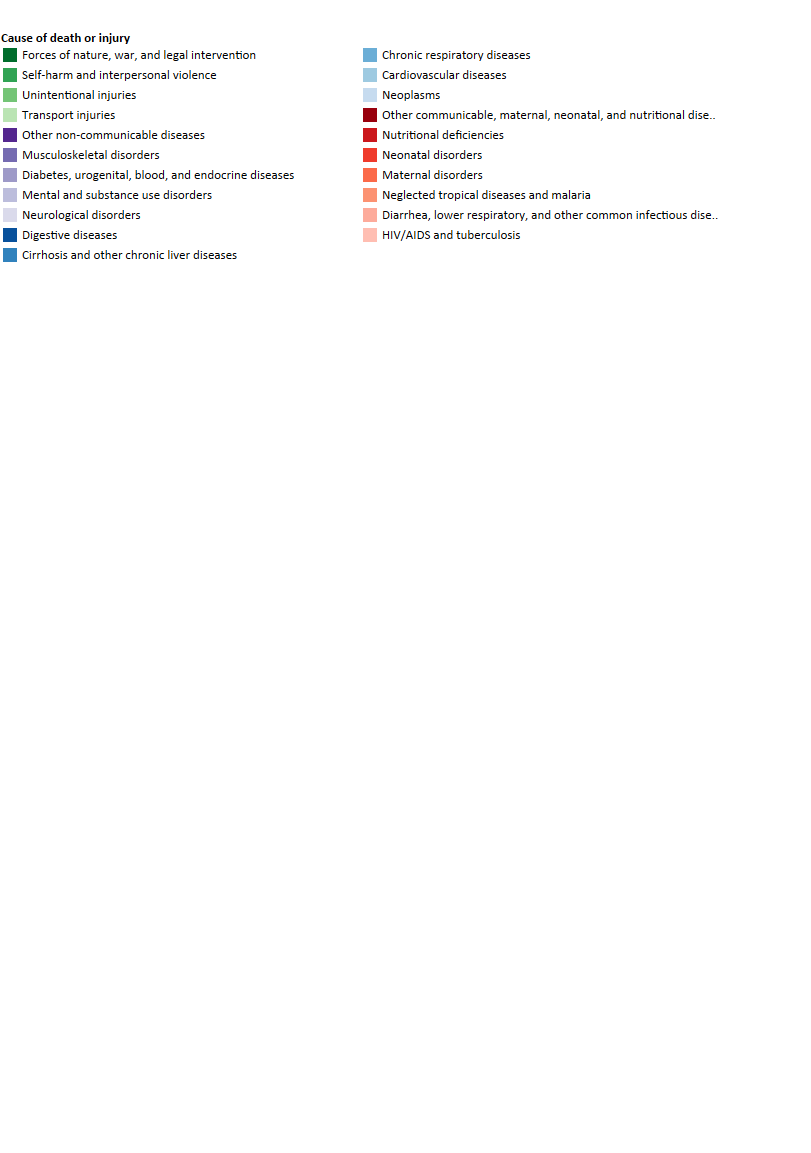


**e-Figure 3 - Key health risks for adolescents in the Eastern Mediterranean Region, in 2015. (Global Burden of Disease Study 2015, Eastern Mediterranean Countries, 2015).**

This figure shows the prevalence of daily tobacco smoking, overweight and obesity, and binge alcohol drinking for adolescents in the Eastern Mediterranean Region in 2015. For each risk, countries are ordered from lowest to greatest prevalence based on males.


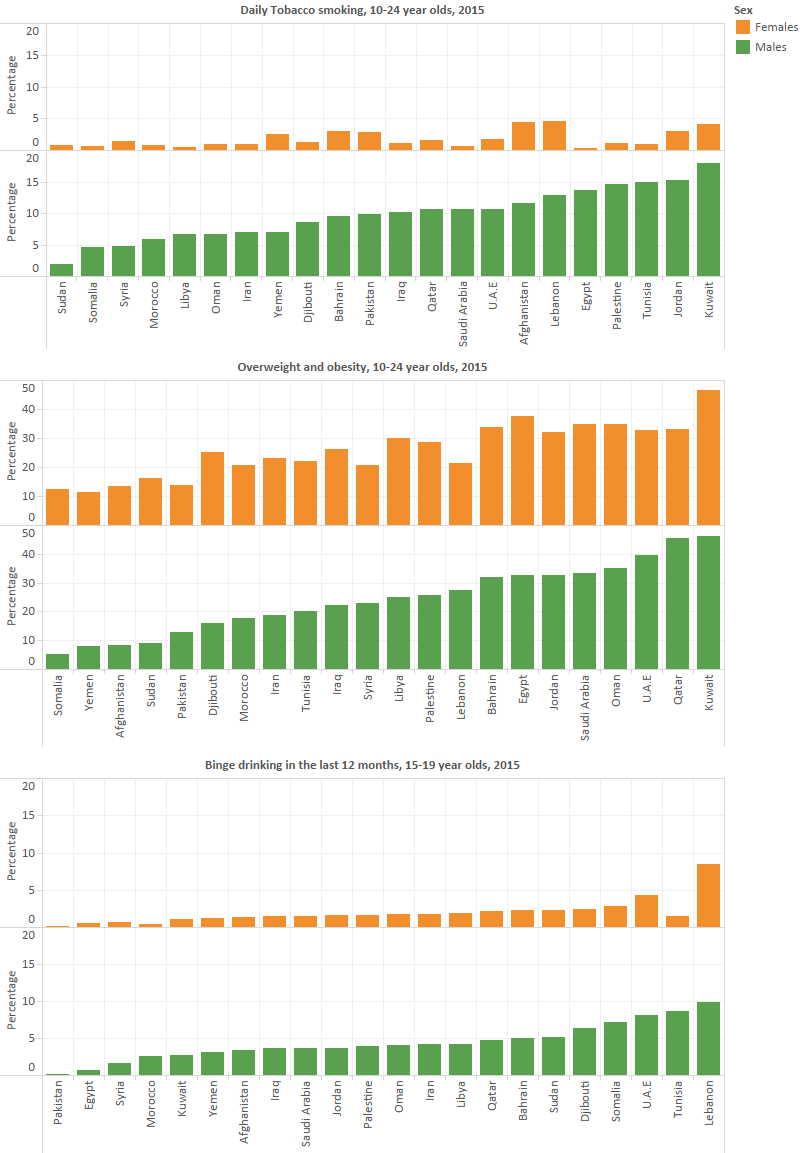


**e-Figure 4 - Key health determinants for adolescents in the Eastern Mediterranean Region, 1990-2015. (ILO, DHS, MICS, Global Burden of Disease Study 2015, Eastern Mediterranean Countries, 1990-2015).**

This figure shows mean education attainment (data: GBD 2015), youth unemployment (data: ILO), unmet need for contraception (data: DHS and MICS) and adolescent fertility rate (data: GBD 2015). For each determinant, countries are ordered from lowest to greatest prevalence based on males.


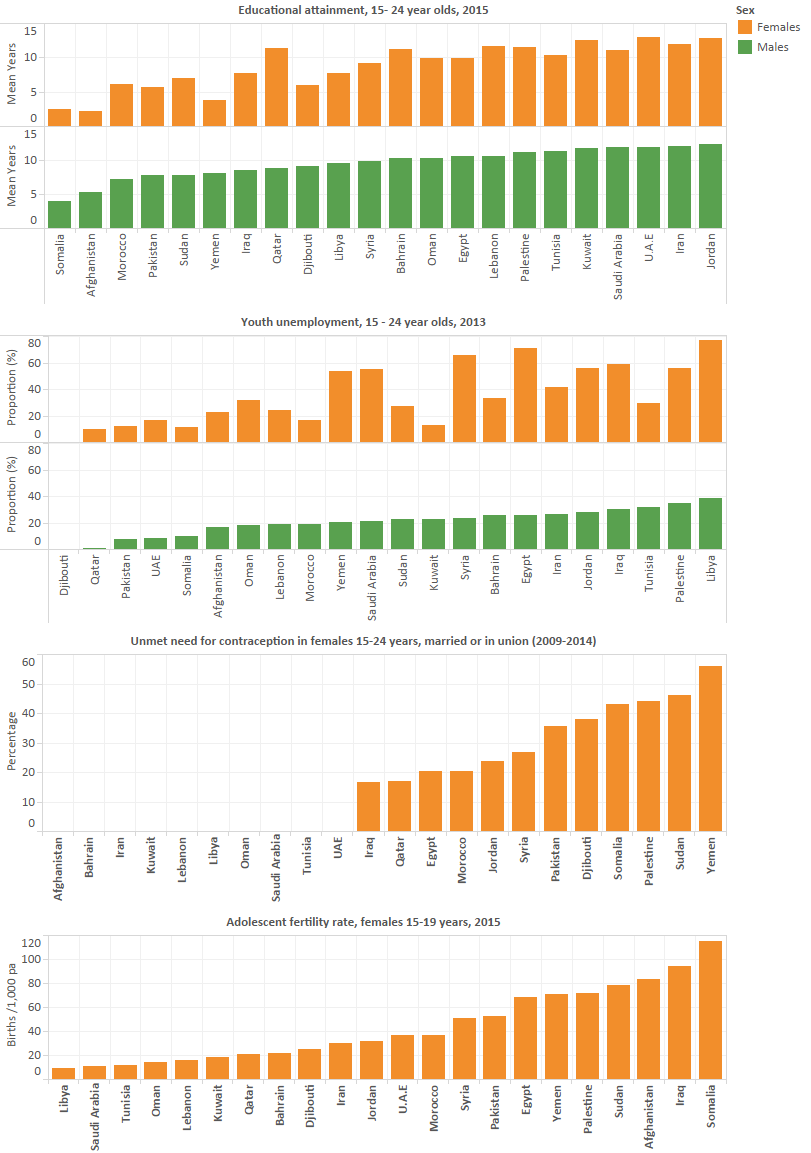


No data available
